# Measurement of Dioxin Emissions from a Small-Scale Waste Incinerator in the Absence of Air Pollution Controls

**DOI:** 10.3390/ijerph16071267

**Published:** 2019-04-09

**Authors:** Gang Zhang, Xiangxuan Huang, Wenbo Liao, Shimin Kang, Mingzhong Ren, Jing Hai

**Affiliations:** 1 Engineering Research Center of None-food Biomass Efficient Pyrolysis and Utilization Technology of Guangdong Higher Education Institutes, Dongguan University of Technology, Dongguan 523808, China; zhanggang@dgut.edu.cn (G.Z.); huangxiangx@dgut.edu.cn (X.H.); liaowenbo110@163.com (W.L.); 2 South China Institute of Environmental Sciences, Ministry of Ecological Environment, Guangzhou 510000, China; renmingzhong@scies.org (M.R.); haijingzg@163.com (J.H.)

**Keywords:** polychlorinated dibenzo-*p*-dioxin and dibenzofuran, emission factor, individual exposure, reduction, small waste incinerator

## Abstract

Polychlorinated dibenzo-*p*-dioxin and dibenzofuran (PCDD/Fs) emissions from basic small-scale waste incinerators (SWI) may cause health risks in nearby people and are thus subject to stringent regulations. The aim of this study was to evaluate PCDD/F emission and reduction of a basic SWI in the absence of air pollution controls (APCs). The results indicated that the stack gas and fly ash presented average PCDD/F levels and emission factors of 3.6 ng international toxic equivalent (I-TEQ)/Nm^3^ and 189.31µg I-TEQ/t and 6.89 ng I-TEQ/g and 137.85µg I-TEQ/t, respectively, much higher than those from large municipal solid waste incinerators (MSWI). PCDD/Fs congener fingerprints indicated that de novo synthesis played a dominant role in the low-temperature post-combustion zone and increased the presence of high-chlorine substituted congeners. On the basis of the emission factor 327.24 µg I-TEQ/t-waste, approximately 3000 g I-TEQ dioxins might be generated in total through basic SWIs and open burning. After refitting an SWI by adding activated carbon injection with a bag filter (ACI+BG), the PCDD/F emissions decreased to mean values of 0.042 ng I-TEQ/Nm^3^, far below the standard of 0.1 ng I-TEQ/Nm^3^, and the removal efficiency reached 99.13% in terms of I-TEQ. Therefore, it is entirely feasible to considerably reduce PCDD/F emissions by refitting basic SWI, which is positive for the future development of rural solid waste (RSW (RSW) disposal by SWI.

## 1. Introduction

Polychlorinated dibenzo-*p*-dioxin and dibenzofuran (PCDD/Fs) are commonly known as dioxins and have become a global environmental problem due to their high toxicity and injurious biological effect [1,2,3]. PCDD/Fs originate from different sources, including waste incinerations, chemical manufacturing, steel mills, vehicles, and accidental landfill fires [4,5]. Identifying potential PCDD/F sources is an essential key step to determine the kind of sources that should be prioritized when implementing a strict emission control.

As the largest developing country, China has constructed about 100 large municipal solid waste incinerators (MSWIs) in large and medium-sized cities by the year 2014. Thanks to remarkable advances in combustion and air pollution control technologies in recent years, PCDD/F emissions from these large MSWIs can be effectively controlled below Chinese environmental standard (0.1 ng international toxic equivalent (I-TEQ)/Nm^3^). With the continuous improvement of rural living standards, the amount of rural solid waste (RSW) is also increasing year by year in China. In view of the per capita waste output of 0.5 kg/(person·d), the annual output of RSW in China is about 100 million tons [6]. Because of some restrictive factors such as scattered population, inconvenient road traffic, high cost of solid waste transportation, insufficient expenditure of waste disposal, and so on, it is difficult to govern the pollution from RSW [7,8]. Small solid-waste heat-treatment facilities such as incinerators and gasifiers have advantages such as the high reduction of waste weight and volume, low investment and construction costs, flexibility of site selection, and so on, which have been widely used in China southwest mountainous area [9]. However, because of the irregular management, unstable operating conditions, and nonexistent air pollution controls (APCs) in a large number of small-scale waste incinerators (SWIs), the emission concentration of PCDD/Fs in the flue gas is relatively high. Chen et al. [10] reported that 11 SWIs in Taiwan emitted 121 µg of PCDD/DFs per ton of waste, approximately 200 times higher than the amount emitted from 21 large MWIs. They concluded that the high value may reflect old age or inadequate air pollution control devices (APCDs) in the SWIs. In Korea, the I-TEQ content of PCDD/Fs ranged from 0.05 to 609.27 ng I-TEQ/Nm^3^, and the average EF_air_ was 1302.4 µg I-TEQ/t from 50 SWIs [11]. Yoo et al. [12] concluded that many SWIs lack effective controls on air pollutants owing to irregular waste feeding, uncontrolled combustion, and insufficient gas cooling systems or APCDs. In addition, Nakao et al. [13] reported that the law has been regulated to forbid the use of SWIs (combustion capacity <50 kg/h) in Japan. They stressed that high attention should be paid to SWIs because of the high amounts of PCDD/F emissions during the incineration of waste with chlorine-containing plastics or copper. These SWIs are an obvious and significant emission source of PCDD/Fs.

Therefore, the contribution from basic SWIs to the total PCDD/F emissions inventory in China may be significant, but few quantitative fundamental data have been used to assess the potential for emission from basic SWIs in the absence of APCs. According to UNEP (*United Nations Environment Program)*’s “Standardized Toolkit for the Identification and Quantification of Dioxin and Furan Releases” (UNEP Toolkit) [14], small waste incinerators (combustion capacity <500 kg/h) with discontinuous operation and irregular waste feeding usually have bad or nonexistent APCDs. The UNEP Toolkit only proposes a theoretical default emission factor to air (EF_air_) of 3500 µg I-TEQ PCDD/Fs per ton of waste from basic SWI. The fundamental data from this important source should be complemented, to facilitate emissions regulation and source reduction. At present, a detailed evaluation of PCDD/Fs from basic SWI, including emission factor and individual exposure assessment, is fairly limited.

The evaluation of the real pollution situation, including emission factors and individual exposure assessment, could urge the government and people to consider the demolition, reconstruction, and technological updating of these obsolete SWIs. Some measures such as catalytic post-combustion, entrained bed, or fixed-bed adsorption, reported by Everaert et al. [15,16,17] could be used to reduce PCDD/F emissions. Activated carbon injection (ACI) is a most widespread technology to control PCDD/F emissions. Dioxins are absorbed by activated carbon captured in a bag filter (BG), with an average removal efficiency of 95% [18]. For proving its feasibility, the comparison of basic SWI emissions to emissions from a hypothetical SWI equipped with ACI+BG technology should be examined. To validate this technology, it is necessary to confirm that PCDD/F emissions from basic SWI could be significantly reduced via APCs. 

The present study is primarily based on full-scale field measurements in an SWI in the absence of APCs. The objective of this study was to evaluate PCDD/Fs emission factor, formation, individual exposure, and reduction of a basic SWI. The combustion conditions were evaluated by examining the relationship between furnace temperature, O_2_, CO, and CO_2_. The main factors affecting PCDD/Fs formation are discussed. The corresponding congener fingerprints of PCDD/Fs were analyzed to identify the main formation mechanism. Then, the individual exposure and cancer risk were assessed considering a model of the total amount of dioxins. Finally, to examine the extent of PCDD/F emission reduction, PCDD/F emissions were measured from an SWI which had implemented the ACI+BG technology.

## 2. Materials and Methods

### 2.1. Basic Information on the Basic SWI

**Figure 1:**
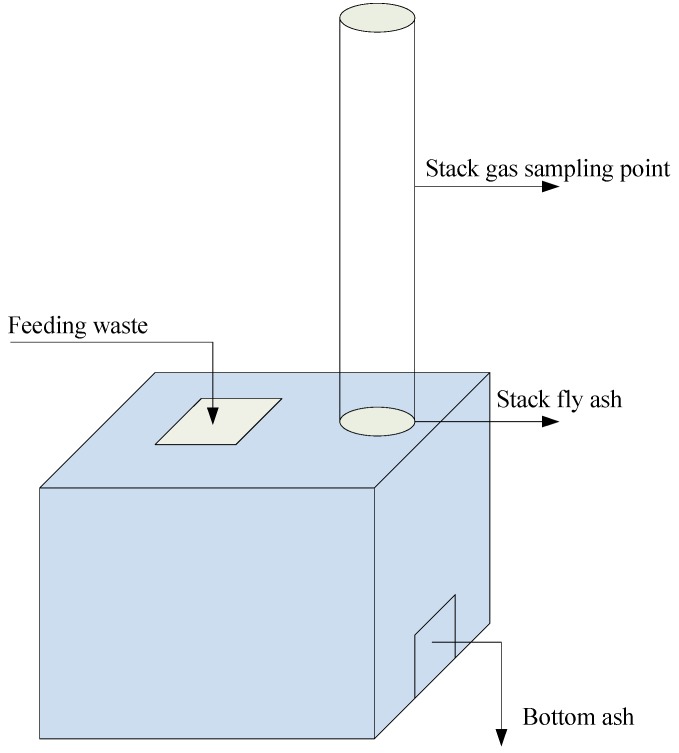
Flow sheet and sampling site of the analyzed small-scale waste incinerator (SWI) in the absence of air pollution controls (APCs).

The investigation was carried out in a basic SWI in a rural area of South China. The SWI had a combustion capacity of 400 kg/hr and was operated 8 h per day. The basic SWI was equipped with a grate and an air inlet at the bottom of the furnace. Waste was fed from the top of the furnace. Combustion air and flue gas for the incinerator are highly dependent on the natural draft. The incinerator was not equipped with APCDs and gas-cooling systems. Thus, the combustion temperature was kept between 500 to 600 °C, and the temperature of the flue gas in the stack was between 200 to 300 °C. Obviously, the SWI was operated in uncontrollable combustion conditions, similar to the open burning condition of solid waste. So, it is appropriate that the SWI was used to analyze the real PCDD/Fs emission factor and formation under uncontrollable combustion conditions of solid waste. Figure 1 shows the flow sheet and sampling site of the basic SWI. In order to validate the proposed solution, the SWI was chosen for our analyses, comparing the results with those of an SWI that implemented the ACI+BG technology.

### 2.2. Waste Characterization

The waste combusted in this study was collected from 10 rural areas located around the basic SWI. To generally describe the type of waste, 10 RSW samples were sampled and analyzed from the 10 rural areas. Table 1 shows an exhaustive characterization of RSW. The values are average values from 10 RSW samples. The ash content was determined through calcination at 850 °C. The calorific value, which amounted to 4278 kJ/kg, was determined by using a WZR-1T-CII microcomputer calorimeter. The low calorific value of the waste can be attributed to its high water and low carbon contents, which were similar to the result reported by Yan et al. [19]. The low calorific value on a dry basis was 10,153 kJ/kg, higher than that on a wet basis. This indicated that decreasing the water content by appropriate pretreatment could increase the calorific value. Kitchen waste comprised the largest fraction of the generated waste (40.25%), reflecting the high content of organics. Combustible materials like plastics and paper were present in relatively high content in accord with the corresponding content of urban waste reported by Zhang et al. [20], which is attributed to the increase of the suburban residents’ income. The heavy metals Cr and Pb mainly derived from plastics. A high content of the plastics results in high contents of Cr and Pb. The main source of Hg was from paper waste in RSW. Cu is mainly linked to electronic and battery waste, dust, rubber, and paper.

### 2.3. Sampling and Analytical Methodologies

To evaluate the formation, emission, and distribution of PCDD/Fs from different components constituting the dioxin inputs and outputs in the SWI, flue gas, stack fly ash, bottom ash, and RSW samples were collected thrice from the corresponding sampling points, amounting to 12 samples. To evaluate the combustion conditions from the basic SWI, the condition parameters including furnace temperature, O_2_, CO, and CO_2_ content were respectively measured nine times during the sampling period. On the basis of the exposure model proposed by Larebeke et al. [21], individual exposure and cancer risk assessments were performed. In order to examine the extent of PCDD/F emission reduction, PCDD/F emissions were measured from the SWI which had performed a technological improvement by adding ACI+BG. The flue gas samples were simultaneously sampled three times at the ACI+BG inlet and outlet, respectively.

The PCDD/F flue gas samples were sampled isokinetically from the stack sampling point with a sampling time ranging between 120 and 180 min, according to American Standard Method EPA 23A. Before flue gas sampling, 13C-labeled sampling standards were spiked into an Amberlite XAD-2 resin. For obtaining representative values, bottom and fly ash samples were simultaneously collected every 30 min until the samples weighed 3 kg. Meanwhile, on the input side, 50 kg of solid waste was taken by the fully mixed multipoint sampling method owing to the general inhomogeneity of the solid waste. The methods used for PCDD/F analysis were adopted from the EPA Method 1613B. These analytical procedures were reported previously, and instrument analysis was conducted using a high-resolution gas chromatograph coupled with a high-resolution mass spectrometer [22,23,24,25]. International toxic equivalent (I-TEQ) values for PCDD/Fs were calculated by the international toxicity equivalency factor (I-TEF).

## 3. Results and Discussion

### 3.1. Evaluation of Combustion Conditions from the Basic SWI in the Absence of APCs

**Figure 2:**
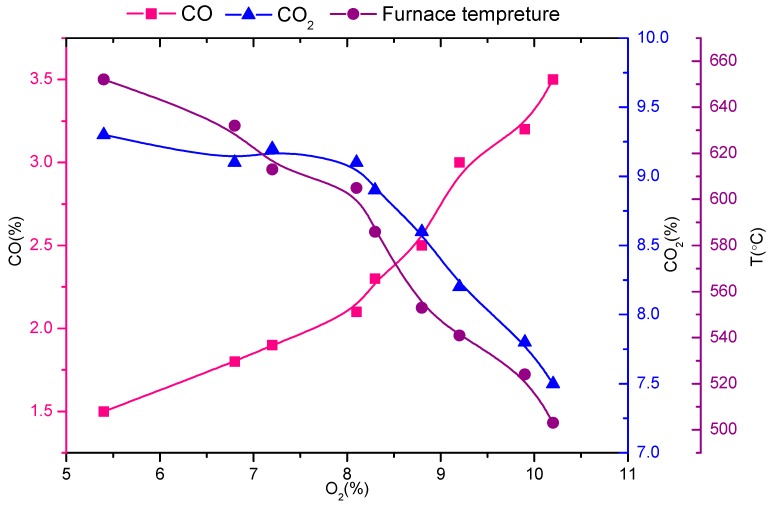
Relationship between furnace temperature, O_2_, CO, and CO_2._

**Figure 3:**
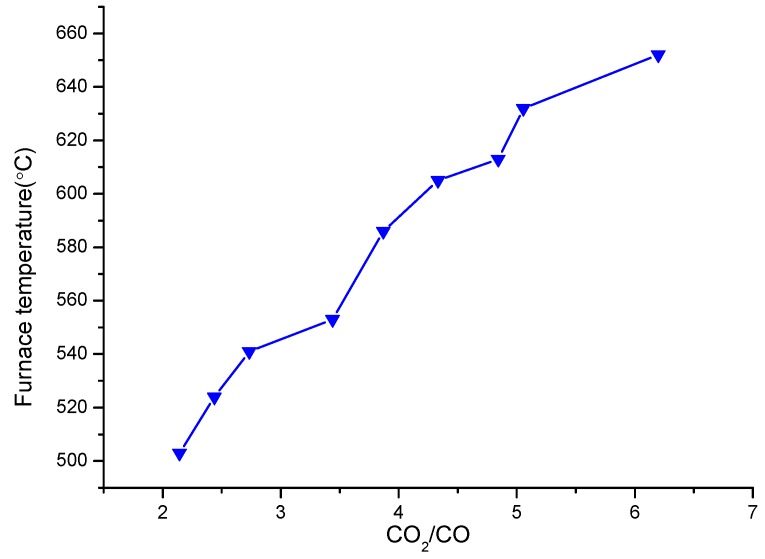
Relationship between furnace temperature and CO_2_/CO ratio.

The control of stable combustion conditions in waste incinerators has become a current concern for decreasing the total PCDD/F emission from waste. The CO value is one of the parameters reflecting the quality of combustion. A higher CO content means that lower amounts of CO_2_ are emitted, and the operation conditions are more unstable. The furnace temperature ranged between 500 °C and 700 °C, far below 850°C which is the normal operating temperature of municipal solid waste incinerators. It is thus clear that the combustion of solid waste was not complete. As shown in Figure 2, the furnace temperature together with O_2_, CO, and CO_2_ varied distinctively in such a manner that as the furnace temperature and CO_2_ level increased, O_2_ and CO levels decreased during waste combustion. O_2_ was recorded between 5 and 11%. As the CO content increased from 1.5% to 3.5% and the CO_2_ content decreased from 9.3% to 7.5%, the furnace temperature decreased from 652°C to 503°C. The higher the amount of CO emitted, the lower the amount of CO_2_ and the more unstable the combustion of solid waste. Therefore, the relationship between furnace temperature and CO_2_/CO ratio was examined (see Figure 3). As the CO_2_/CO ratio gradually increased, the furnace temperature obviously increased. This confirmed that the CO_2_/CO ratio can be used as an indicator of solid waste combustion conditions.

### 3.2. Content and Formation of PCDD/Fss from the Basic SWI in the Absence of APCs 

Table 2 shows PCDD/F emission concentrations and I-TEQ values in the stack gases, stack fly ashes, and bottom ashes of the SWI. The stack flue gas presented a mean level of 3.6 ng I-TEQ/Nm^3^, much higher than the legal upper limit (0.1 ng I-TEQ/Nm^3^) permitted in emissions from large MSWI. The I-TEQ value of the stack gas was of the same order of the values from an unregulated small incinerator [2]. The stack fly ash samples also presented high mean levels corresponding to 6.89 ng I-TEQ/g, which exceeded the Japanese environmental quality standards for soil (less than 1 ng I-TEQ/g) [26]. Therefore, the fly ash from SWI should be classified as hazardous waste and require special treatment. However, the mean PCDD/F content from bottom ash was 0.37 ng I-TEQ/kg, much lower than that from fly ash. The bottom ash might be applied for constructions blocks or other building materials. These results indicate that SWIs emit dioxin at much higher concentrations than large MWIs for two reasons. On one hand, a high amount of PCDD/Fs was formed through the whole operation process as a consequence of the worst combustion and flue gas treatment conditions attributed to discontinuous waste combustion, open gates during waste feeds, poorly performing combustion chambers, and so on. The specific factors affecting PCDD/Fs formation are considered in the last section. On the other hand, the SWI was totally unable to control PCDD/Fs emissions without any gas-cooling facility and APCs, leading to the greatest amount of dioxin emissions. Thus, the PCDD/Fs formation degree, in the case of practical solid waste incineration, can be well determined by using just the dioxin emission factor of the input (solid waste) and the output (stack gas, fly ash, and bottom ash) in the SWI.

Table 2 also shows PCDD/F levels in the RSW samples. The average content was approximately 2.58 ng I-TEQ/kg, slightly lower than that reported by Zhang et al. [22] (China: 15.56 ng I-TEQ/kg), Abad et al. (Spain: 4.4–13.3 ng I-TEQ/kg) [27], and Makoto et al. (Japan: 1.3–16 ng I-TEQ/kg) [28]. The PCDD/F content from solid waste is generally very low, attributed to the fact that many countries have made great efforts to reduce the global PCDD/F emissions. In USA, the controlled sourced emissions decreased from 14.0 kg I-TEQ PCDD/Fs in 1987 to 0.6 kg in 2012 [18]. The dioxin emissions from waste in energy plants were also reduced from 10.2 kg I-TEQ in 2004 to 6 kg in 2008 in China [18]. In 2014, the dioxin emission standard was reduced from 1 ng I-TEQ/m^3^ to 0.1 ng I-TEQ/m^3^.

The factors significantly affecting PCDD/Fs formation can be subdivided into two categories: (1) operating factors and (2) chemical and catalytic factors. Poor combustion conditions appear as inadequate temperature, residence time, and turbulence (“3T”), resulting in a mix of incomplete combustion products. These poor operating conditions result from ‘bad’ waste, outdated incineration technology, and bad operational management. These poor operating conditions would lead to higher levels of PCDD/Fs. The RSW contained a certain amount of kitchen waste, many plastic bags, and inappropriate solid waste with a high content of chlorine and metal copper, leading to increased PCDD/Fs formation in the incineration process. In terms of chemical and catalytic factors, the presence of metals provided the catalytic conditions required to fix chlorine on carbon structures and oxidize precursor compounds, producing more PCDD/Fs. Catalytic metals, of which Cu is the most representative, are associated with particulates. Many researchers [29,30,31] have confirmed that the addition of Cu as a catalyst promotes the formation of PCDD/Fs. Previous studies [32,33] have examined the reactivity of different chlorine compounds and found the reactivity order was KCl<CaCl_2_<FeCl_3_<<CuCl_2_. In this work, 35.34 mg/kg Cu was detected in the RSW, as shown in Table 1. It is evident that Cu in the RSW should be an important factor for high dioxin emissions. On the other hand, sulfur could inhibit dioxin formation, and the suppression effect of S-containing inhibitors on dioxin formation has been reported [34].

### 3.3. Congener Distribution and Fingerprint Analysis of PCDD/Fs from the Basic SWI

**Figure 4:**
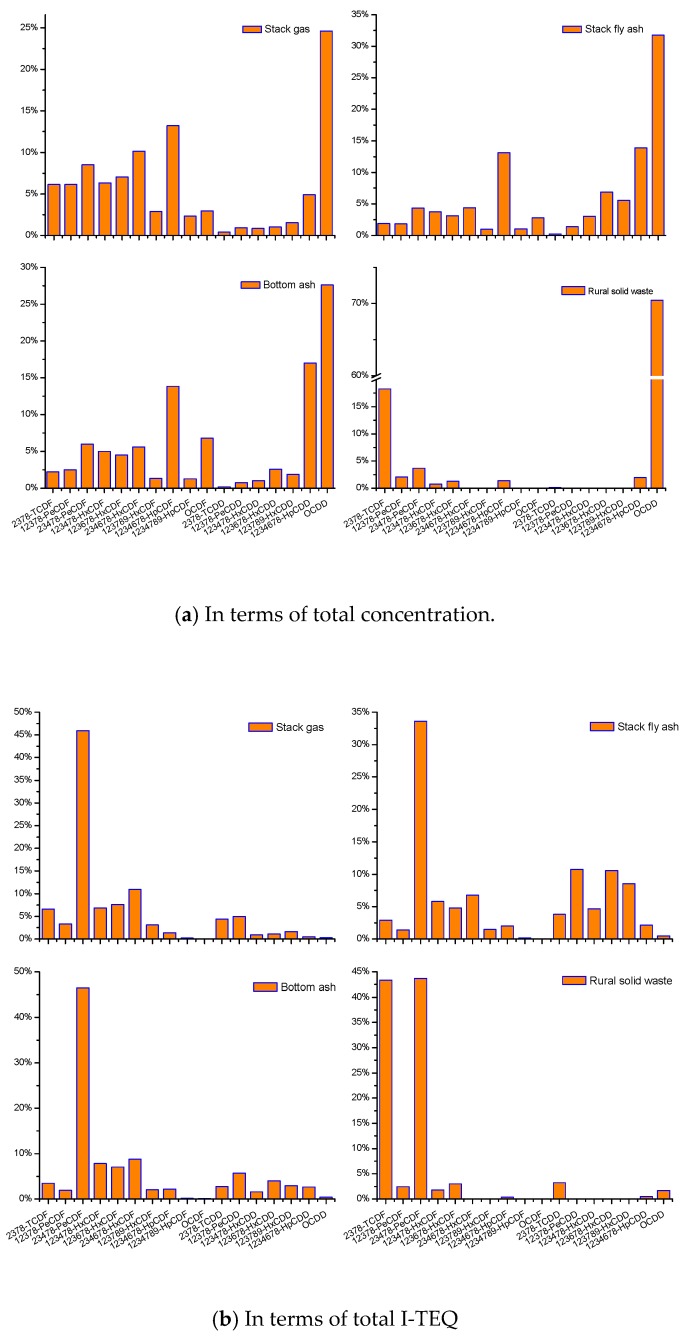
PCDD/F congener profiles in stack gas, fly ash, bottom ash, and RSW in terms of total concentration (**a**) and in terms of total I-TEQ (**b**).

The PCDD/F congener profile is often used as a fingerprint or signature. The fingerprinting of PCDD/Fs has been widely adopted in source identification and formation mechanism elucidation [24]. Figure 4a shows the relative congener profiles (in terms of total concentration) in stack gas, fly ash, bottom ash, and RSW samples. In the output side, the stack gas, fly ash, and bottom ash presented certain similarities. With an increasing chlorinated level, the content of the 2,3,7,8-PCDD congener increased, but the content of 2,3,7,8-PCDF congener showed irregularities. With regard to PCDDs, the stack gas, fly ash, and bottom ash produce the OCDD (octachlorodibenzo-*p*-dioxin) congener as a major constituent corresponding to about 25–35% of total PCDD/Fs, followed by 1,2,3,4,6,7,8-HpCDD. As for PCDFs, the 1,2,3,6,7,8-HpCDF was produced as a main congener, followed by 2,3,4,6,7,8-HxCDF and 2,3,4,7,8-PeCDF. These profiles are consistent with the reported emission patterns from MSWI [22,35]. The congener distribution was in line with the PCDD/F fingerprints, suggesting the dominant role of de novo synthesis reported by Everaert et al. [36]. This indicates that de novo synthesis played a dominant role in the low-temperature post-combustion zone of the basic SWI. By contrast, the congener profiles of RSW in the input side differed remarkably from those in the output side. The RSW concentration congener profiles were clearly dominated by a high content of OCDD, followed by 2,3,7,8-TCDF. The fractions of OCDD and 2,3,7,8-TCDF reached around 90% of the total PCDD/Fs, which is similar to the profile reported by Abad et al. [27]. Besides, the concentration of all 2,3,7,8-congeners from the output samples were much higher than those from the input solid waste sample. PCDD/Fs from the feeding waste were not decomposed in the furnace, because the furnace temperature (500–600 °C) was lower than 850 °C. This difference between input side and output side indicates that all 2,3,7,8-congeners were first heavily synthesized through high-temperature gas-phase reactions in the high-temperature combustion zone and then, through the dominant de novo mechanisms, in the low-temperature post-combustion zone from the SWI.

For PCDD, the TEQ-equivalence of 1234678-HpCDF, 1234789-HpCDF, and OCDF was the lowest. For PCDF, the TEQ-equivalence of 1234678-HpCDD and OCDD was the lowest. The high chlorine substituted-PCDD/F, with the lowest TEQ-equivalence factor, was mostly generated by de novo synthesis. As shown in Figure 4a, the high chlorine substituted-PCDD/F, with the lowest TEQ-equivalence factor, was mostly generated by de novo synthesis. The extra presence of these congeners well-matched the fingerprints of de novo synthesis. Two major mechanisms have been proposed, i.e., precursor formation and de novo synthesis. Everaert et al. [36,37] explained why the role of precursor formation is not obvious in the actual incineration process. The overestimation of precursor formation occurs in most laboratory-scale investigations, where precursor concentrations of 10^5^−10^6^ µg/Nm^3^ are used at temperatures ≥ 300 °C and solid residence time of 2–60 min. In the actual waste incinerator, the precursor concentration is less than 10 µg/Nm^3^, and the residence time is less than 1 min. Hence, precursor formation is severely overestimated in laboratory-scale investigations. The precursor formation yield of the investigation was largely overestimated by a factor of (experimental concentration/real concentration) ^1.5^ = (10^5^–10^6^ µg/Nm^3^/10 µg/Nm^3^) ^1.5^ = 10^6^–10^8^ [36]. De novo synthesis requires carbon and O_2_, the concentrations of which are much higher than those of the precursor. The stack gas temperature has a very strong influence on PCDD/Fs formation. The stack gas temperature was between 200 and 300°C, a temperature window where the PCDD/F de novo synthesis reaction is extremely sensitive. Considering the overestimated rate of precursor synthesis, it is clear that de novo synthesis was several orders of magnitude faster than the precursor reaction, confirming the extra presence of some of the congeners (with the lowest TEQ-equivalence factor).

Figure 4b correspondingly shows the congener profiles in terms of total I-TEQ. The stack gas, fly ash, and bottom ash also presented certain similar congener profiles. These profiles were mainly characterized by 2,3,4,7,8-PeCDF, accounting for approximately 40–50% of the total I-TEQ PCDD/Fs. This congener distribution was identified as the representative characteristic of the PCDD/Fs emission during the incineration of MSW, PVC, textile waste, and sewage sludge [38]. The results indicate that the dioxin emission profiles were not affected by discontinuous waste combustion, open gates during waste feeds, and poorly performing combustion chambers.

### 3.4. Emission Factor, Mass Balance, and Individual Exposure Assessment of PCDD/Fs from the Basic SWI

**Figure 5:**
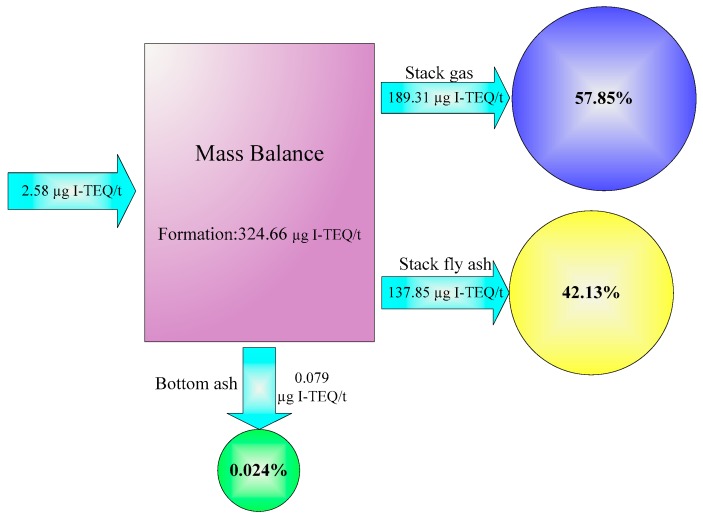
PCDD/F emission factor, distribution, and mass balance in the SWI. The percentage (%) is the proportion of individual output to total output.

With the purpose of evaluating the real situation of the SWI, the PCDD/F mass balance from input to output was investigated. Figure 5 shows the PCDD/F emission factor, distribution, and mass balance across the whole SWI. In the output balance, three forms of matrixes (stack gas, fly ash, and bottom ash samples) over the three sampling campaigns yielded three average values relying on a total of nine values. The PCDD/F emission factors in bottom ash, stack fly ash, and stack gas were 0.079 ± 0.011, 137.85 ± 12.41 and 189.31 ± 9.43 µg I-TEQ/t-waste, respectively. A total emission factor of 327.24 µg I-TEQ/t-waste was calculated, much higher than that resulting from the large MSWI (22 µg I-TEQ/t-waste) [22]. The higher emission burden of PCDD/Fs from the SWI was closely linked to higher emission factors. In accordance with PCDD/F percentage distribution, the PCDD/F was distributed almost equally between stack gases (approximately 57.85%) and stack fly ashes (approximately 42.13%), whereas the bottom ashes contributed a minor fraction to the total PCDD/F output (approximately 0.024%) as shown in Figure 5. This result is different from that reported by previous research [22] in a modern MSWI. The previous distribution characteristics showed that fly ashes contributed approximately 85% to the total output, and stack gas contributed merely 1%. The difference should be ascribed to the injection of activated carbon for PCDD/F removal from the APCs of MSWI, which generated PCDD/F-enriched fly ash. As shown in Figure 5, the PCDD/F input emission factor was 2.58 µg I-TEQ/t-waste, much lower than the total output value (327.24 ± 21.86 µg I-TEQ/t-waste), suggesting a positive PCDD/F balance of 324.66 µg I-TEQ/t-waste. In the sampling campaign, the positive balance indicated that the SWI was a source of PCDD/Fs. Because PCDD/Fs presented in the waste were not destroyed when the combustion temperature was lower than 850 °C, it can be inferred that 324.66 µg I-TEQ PCDD/Fs were formed via incineration of one ton (wt) of solid waste by heterogeneous catalytic reactions.

**Figure 6:**
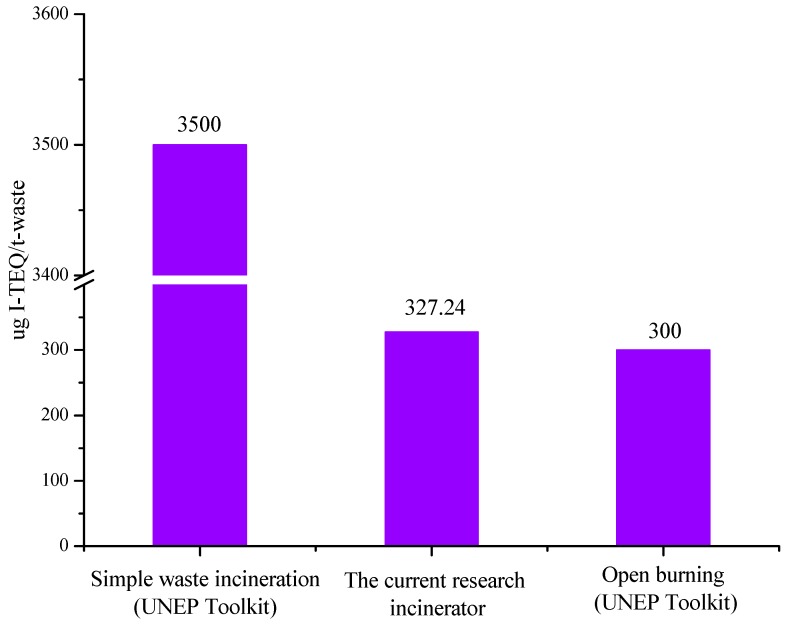
Emission factors from basic waste incineration (UNEP Toolkit), the current research incinerator, and open burning (UNEP Toolkit).

The UNEP Toolkit provides a methodology to develop PCDD/Fs release inventories without dioxin measurements by providing default emission factors for a large number of source categories. The emission factor from the SWI in the current study was compared with that from basic waste incineration in the Toolkit, as shown in Figure 6. The Toolkit’s emission factor (3500 µg I-TEQ/t) was one order of magnitude higher than that from the basic SWI (327.24 µg I-TEQ/t). This field measurement result can provide fundamental data for the improvement of the Toolkit in the future. In addition, the emission factor from the SWI was similar to the Toolkit’s emission factor during open burning of waste, as shown in Figure 6. This indicated that the dioxin formation conditions in the SWI were analogous to those during open burning of waste, and the measurement in the SWI can reflect the generation of dioxin in the process of open burning. During open burning, dioxin emissions are related to the development of a fire (flaming vs smoldering), to different combustion parameters (temperature, turbulence, oxygen, etc.), and to other factors influencing dioxins formation, such as chlorine, catalytic metals, soot, and ash [39]. There are few effective methods to collect representative samples. It is fairly difficult to evaluate and quantify dioxin emission from open burning. This results in emission factor values ranging over several orders of magnitude [39]. Therefore, the PCDD/F emissions from open burning may be an important missing source. The emission factor value from the basic SWI could represent that from open burning.

Individual exposure was assessed using the exposure parameters proposed by Larebeke et al. [21]. Table 3 presents a comparison between individual exposure from the Chinese basic SWIs in the absence of APCs or open burning and those from a Belgian dioxin incident. The model assumes an even distribution of the pollution over the Chinese population about 1000 million. About 100 million tons of RSW in China is produced. About 10 million tons of RSW (10% of the total amount) is disposed of by basic SWIs or open burning [7]. On the basis of the emission factor 327.24 µg I-TEQ/t-waste, approximately 3000 g I-TEQ dioxin might be generated through basic SWIs and open burning. The total emission value (3000 g I-TEQ) was higher than that (1757.6 g I-TEQ) from all waste combustion, including MSW, hazardous waste, medical waste, general industrial waste, and metal conductor combustion. Yet the value (3000 g I-TEQ) was lower than that (4666.9 g I-TEQ) from steel and metals production [18]. It is assumed that 1% of this amount was introduced in the food chain, namely, approximately 30 g I-TEQ, 30% of which was ingested by 10 million Chinese. With a mean body weight of 60 kg for the model citizen (children included), this represents an average intake of 150 pg I-TEQ/kg body weight. Similarly, the 3000 g I-TEQ of PCDD/Fs would result in an increase in body burden, with 150 pg I-TEQ/kg body weight. This represents an increase of 2.2%, assuming a mean baseline PCDD/F body burden of 6.88 ng I-TEQ/kg [21]. This value also corresponds to an intake corresponding to 0.006 pg/kg per day over 70 years (approximately 25,000 days). With regard to cancer risk assessment based on the cancer risk estimation of 100 in 100 million for a lifetime exposure of 0.006 pg I-TEQ/kg body weight per day, the incremental exposure, estimated to be equivalent to an intake 0.006 pg/kg per day over 70 years, would signify a risk of 10 additional cancer deaths per 10 million Chinese individuals.

### 3.5. Comparison of Emissions to Those from the Refitted SWI Equipped with APCs

In order to present a solution, PCDD/F emissions from the basic SWI were compared to those from a refitted SWI equipped with ACI+BG. As shown in Table 4, the basic SWI PCDD/F emissions were much higher than those of the refitted SWI. After technical improvements, the PCDD/F emissions decreased to mean values of 0.042 ng I-TEQ/Nm^3^, far below the standard of 0.1 ng I-TEQ/Nm^3^, with the concentration of several congeners even below their respective detection limits. The effect of the ACI+BG technology on PCDD/F emission was fairly significant. The PCDD/F concentration in flue gas before ACI+BG was 35.73 ng /Nm^3^ or 4.83 ng I-TEQ/Nm^3^. The PCDD/Fs removal efficiency is given on ng I-TEQ/Nm^3^ and ng/Nm^3^, respectively. The percentages were calculated as the ratio between the reduction and the inlet value. The removal efficiency reached 99.13% and 98.24% in terms of I-TEQ and concentration, respectively. The removal efficiency was in the range of 93.3–99.4% by entrained-phase adsorption of PCDD/F from incinerator flue gases [17]. This indicated that refitting the basic SWI could considerably lower the PCDD/F emissions. This development would allow efficient RSW disposal by SWI in rural areas.

## 4. Conclusions

The emission and reduction of PCDD/Fs from a basic SWI in the absence of APCs were investigated. The dioxin emissions were much higher than those from MSWIs. In total, approximately 3000 g I-TEQ/year dioxins were generated through basic SWIs and open burning in China, which are an important source of PCDD/Fs. The extra presence of the high-chlorine substituted congeners was facilitated by de novo synthesis in the basic SWI. After refitting an SWI by adding ACI+BG, the PCDD/F emissions met the standard of 0.1 ng I-TEQ/Nm^3^, indicating that ACI+BG is a promising technology for the future development of RSW disposal by SWI.

The study reported in this paper consisted of limited dioxin emission data including flue gas, fly ash, and bottom ash. Considering the complex set of variables that affect dioxin emissions from the basic SWI, further studies focusing on examining the important factors on the dioxin formation and emission are under consideration. The effects of waste composition, chlorine/sulfur content and type, catalytic metals, entwining, and heating value on combustion conditions and dioxin emissions would be revealed. Additionally, the treatment and disposal of fly ash with a high dioxin content should be investigated.

## Figures and Tables

**Table 1:** Rural solid waste (RSW) characterization.

Proximate Analysis			
Moisture content (wt. %)	61.57	Ash (wt. %)	8.91
Volatile matter (wt. %)	26.38	Fixed carbon (wt. %)	3.14
Low calorific value (kJ/kg)	4278	Low calorific value (dry basis, kJ/kg)	10,153
Physical composition analysis (wt. %)			
Kitchen waste	40.25	Grass and wood	3.88
Plastic	21.93	Glass	6.12
Paper	18.54	Metal	0.27
Textile	6.88	Sandy soil	2.13
Elemental analysis on dry basis (wt. %)			
C	35.9	H	4.54
O	25.73	N	0.44
S	0.12	Cl	0.11
Heavy metals on dry basis (mg/kg)			
Pb	20.81	Cr	298.13
Hg	1.74	As	N.D.
Cd	N.D.	Cu	35.34

N.D. means not detected.

**Table 2:** Polychlorinated dibenzo-*p*-dioxin and dibenzofuran (PCDD/F) content in the output and input from the SWI (*n*: sampling times). I-TEQ, international toxic equivalent.

Output/Input	PCDD	PCDF	Total PCDD/F	Total I-TEQ PCDD/F
Output:				
Stack gas (ng/Nm^3^) (*n* = 3)	13.33 ± 0.20	25.57 ± 0.68	38.90 ± 0.88	3.60 ± 0.18
Stack fly ash (ng/g) (*n* = 3)	66.51 ± 3.00	39.52 ± 0.39	106.02 ± 3.40	6.89 ± 0.62
Bottom ash (ng/kg) (*n* = 3)	2.92 ± 0.29	2.81 ± 0.19	5.73 ± 0.48	0.37 ± 0.05
Input:				
RSW (ng/kg) (*n* = 3)	44.33 ± 2.96	16.78 ± 0.93	61.11 ± 3.89	2.58 ± 0.06

**Table 3:** Comparison between individual exposure from the Chinese basic SWIs or open burning and that from a Belgian dioxin incident.

Comparison between Individual Exposure	Dioxin Exposure Data from the Chinese Basic SWIs or Open Burning	Exposure Data from the Belgian Dioxin Incident [21]
Total amount	3000 g I-TEQ	1 g I-TEQ
Average intake	150 pg I-TEQ/kg body weight based on 1000 million Chinese	500 pg I-TEQ/kg body weight based on 10 million Belgians
Intake per day over 70 years	0.006 pg I-TEQ/kg per day	0.02 pg I-TEQ/kg per day
Rate of increase based on mean dioxin body burden	2.2%	7%
Cancer risk assessment	10 additional cancer deaths/10 million Chinese	32 additional cancer deaths/10 million Belgians

**Table 4:** Comparison with PCDD/F emissions from the refitted SWI (*n*: sampling times).

Comparison with PCDD/F Emissions	PCDD	PCDF	Total PCDD/F	I-TEQ Total PCDD/F
Stack gas from the basic SWI (ng/Nm^3^) (*n* = 3)	13.33 ± 0.20	25.57 ± 0.68	38.90 ± 0.88	3.60 ± 0.18
Flue gas at the ACI+BG inlet from the refitted SWI (ng/Nm^3^) (n = 3)	16.71 ± 1.51	19.0 2±2 .50	35.73 ± 4.01	4.83 ± 0.56
Flue gas at the ACI+BG outlet from the refitted SWI (ng/Nm^3^) (n = 3)	0.299 ± 0.035	0.304 ±0 .013	0.604 ± 0.022	0.042 ± 0.005
PCDD/Fs removal efficiency by ACI+BG (%)			98.24%	99.13%
